# Developing pathways to clarify pathogenicity of unclassified variants in Osteogenesis Imperfecta genetic analysis

**DOI:** 10.1002/mgg3.912

**Published:** 2019-09-30

**Authors:** Meena Balasubramanian, Emma Hobson, Mars Skae, Janine McCaughey, David J. Stephens

**Affiliations:** ^1^ Highly Specialised Severe, Complex & Atypical OI Service Sheffield Children’s NHS Foundation Trust Sheffield UK; ^2^ Sheffield Clinical Genetics Service Sheffield Children's NHS Foundation Trust Sheffield UK; ^3^ Academic Unit of Child Health University of Sheffield Sheffield UK; ^4^ Yorkshire Clinical Genetics Service Chapel Allerton Hospital Leeds UK; ^5^ Department of Paediatric Endocrinology Royal Manchester Children’s Hospital Manchester UK; ^6^ School of Biochemistry, Faculty of Life Sciences University of Bristol Bristol UK

**Keywords:** fractures, genetic analysis, osteogenesis imperfecta, targeted gene panel testing, Variant of uncertain significance

## Abstract

**Background:**

With increased access to genetic testing, variants of uncertain significance (VUS) where pathogenicity is uncertain are being increasingly identified. More than 85% Osteogenesis Imperfecta (OI) patients have pathogenic variants in *COL1A1/A2*. However, when a VUS is identified, there are no pathways in place for determining significance.

**Objective:**

Define a diagnostic pathway to confirm pathogenicity, providing patients with definitive genetic diagnosis, accurate recurrence risks, and prenatal testing options.

**Methods:**

Functional studies on collagen secretion from cultured patient fibroblasts combined with detailed phenotyping and segregation family studies.

**Results:**

We demonstrate data from a family with a VUS identified in type I collagen.

**Family‐1:**

Six‐year‐old boy with failure‐to‐gain weight, talipes, fractures, on and off treatment with Pamidronate as diagnosis of OI uncertain. Transiliac bone biopsy at 2 years of age demonstrated active new bone formation within periosteum; bone cortices were normal thickness but increased porosity. Trabecular bone showed features of advanced osteoporosis. Genetic testing identified a de novo* COL1A1* c.206_208delTGT, p.Leu69del variant. Sibling with similar phenotype but no fractures as yet, tested positive for variant raising concerns regarding her diagnosis, and management.

Results from three independent experiments (cell immunofluorescence, collagen secretion assay by Western Blot, and unbiased proteomics) from cultured patient fibroblasts demonstrate COL1A1 c.206_208delTGT, p.Leu69del variant causing a substantial defect to collagen extracellular matrix assembly confirming variant pathogenicity.

**Conclusion:**

Access to genetic testing in OI is increasing as advances in genetic technologies decreases cost; a clinical diagnostic pathway needs to be implemented for managing variants identified by such testing.

## Graphical abstract

Inherited bone fragility disorders, of which Osteogenesis Imperfecta (OI) is the commonest, are rare (1 in 15,000). In this study, we defined ways to clarify pathogenicity of a variant of uncertain significance leading to bone fragility using advanced sequencing technologies and to characterize the biological consequences of these mutations using advanced molecular cell biology.

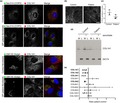

## 1 INTRODUCTION

Inherited bone fragility disorders, of which Osteogenesis Imperfecta (OI) is the commonest, are rare (1 in 15,000) (Lim, Grafe, Alexander, & Lee, 2017). However, they are complex, require a multidisciplinary approach, consume significant resources, and often go unrecognized outside specialized settings, increasing patient burden. Bone fragility is commonly perceived as a disorder of the elderly, due to degenerative osteoporosis. However, recent advances in understanding of pathways relevant to treatment of osteoporosis have come from studies of inherited bone fragility in multigenerational families. OI is used to describe inherited bone fragility disorders in childhood; mutations in 15 genes affecting type I collagen formation, processing and stability, or osteoblast function have now been described. Over 85% of patients with OI have pathogenic variants in type 1 collagen genes (*COL1A1/A2*) (Forlino & Marini., 2016); the importance of type 1 collagen is underscored by the fact that collagen is the most abundant protein in the human body. Although, treatment is available for OI in the form of bisphosphonates, this does not improve the quality of bone or cure the condition.

Osteogenesis Imperfecta has historically remained a clinical diagnosis in most cases due to costs and limited access to genetic testing. However, due to the need to provide patients and their families with accurate diagnosis, information on prognosis, and reproductive options, genetic testing became increasingly part of their clinical work‐up. With reducing costs of genetic testing and wider access to whole exome/ genome sequencing studies, more patients with mild‐moderate OI access genetic testing. Although, this is a positive development for families concerned, this does bring about unclassified variants, more frequently referred to as Variants of Uncertain Significance (VUS) where the significance of a variant to assign causality is uncertain that needs interpreting.

In this study, we defined ways to clarify pathogenicity of a VUS leading to bone fragility using advanced sequencing technologies and to characterize the biological consequences of these mutations using advanced molecular cell biology. Patient samples were analyzed to define the extracellular matrix (ECM) deposited by patient fibroblasts. We also defined the effect of a pathogenic variant (in procollagen encoding genes) on cell structure and function. Specifically, procollagens present a problem to cells in terms of both the abundance and potentially because of their physical size (Malhotra & Erlmann, 2015). Adaptation of the canonical secretory pathway machinery is thought to underpin effective procollagen secretion (Malhotra & Erlmann, 2015). We planned to define VUS in *COL1A1* using a family encountered in clinical practice as an exemplar and determine how they affect trafficking of procollagen through the secretory pathway with a goal of defining mechanisms that might be targeted to modulate procollagen secretion and ECM assembly.

There is increasing evidence that the composition of the ECM directly affects cell structure and function. We have recently found evidence for a potential feedback mechanism by which ECM controls the synthesis of nascent ECM glycoproteins (Stevenson et al., 2017). Here, we exploited the differences in patient cell‐derived matrix to determine how this affects further synthesis, secretion, and assembly or the glycoprotein‐rich ECM. This model suggested that cells have the capability to adapt to defective ECM and that further modulation of this capacity could be a worthwhile target for clinical intervention.

## 2 CLINICAL REPORT

### 2.1 Family 1

The proband (Patient 1a) was the first child of healthy, nonconsanguineous White European parents with no significant family history. The pregnancy was complicated by hyperemesis but otherwise normal. Patient 1a was born at term with 2.57 kg (0.4th–2nd centile) and was in a good condition immediately after birth. He was noted to have bilateral cryptorchidism and under‐virilized male genitalia with a 46, XY karyotype. He required three surgeries in the first year of life for testicular descent. He was also noted to have bilateral calcaneus talipes equinovarus needing Ponseti correction. He developed fragility fractures, initially of his right femur, a transverse fracture at the top of the Ponseti above knee casting; then right proximal tibia with resultant nonunion and a fracture of the distal tibia identified incidentally. He was commenced on treatment with Pamidronate at 14 months of age which was discontinued briefly and recommenced until 40 months of age.

**Figure 1:**
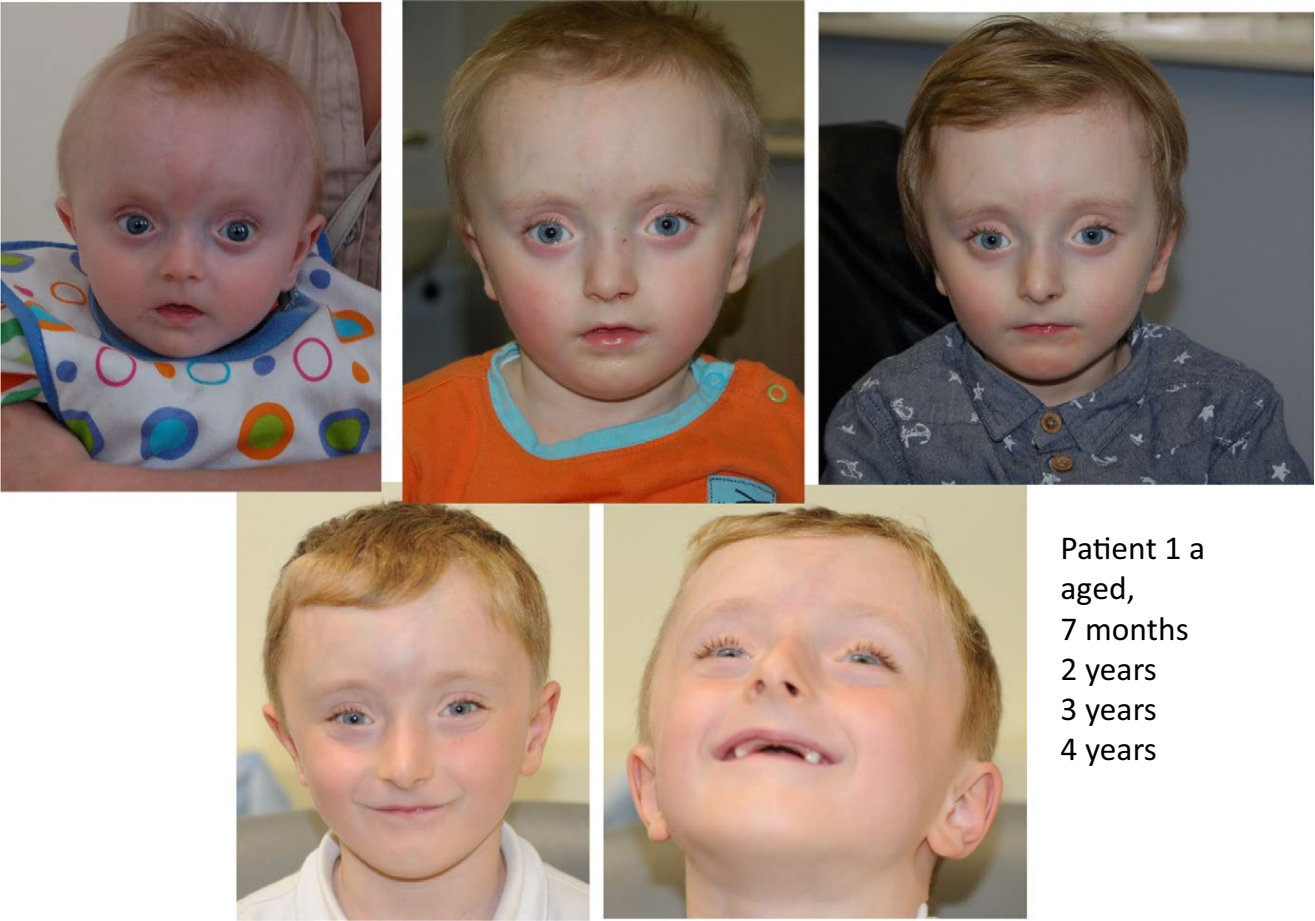
Facial dysmorphism in Patient 1a (aged 7 months, 2 years, 3 years, and 4 years) with small pinched nose, small mouth with thin upper lip, and prominent veins over forehead with brachycephaly, blueish tinge to sclerae, and dental enamel hypoplasia

Patient 1a is currently 7 years of age and presents with significant short stature (with height consistently just below the 0.4th centile, weight on the 0.4th centile and head circumference between 0.4th–2nd centiles). He has normal motor development with mild speech delay and possible concerns regarding attention deficit hyperactivity disorder for which he is being assessed. On examination, he has pale skin with prominent veins over his forehead, brachycephaly, high anterior hairline, blueish tinge to his sclerae, small pinched nose, small mouth with thin upper lip, and a small jaw. Figure 1 shows evolution of his facial dysmorphism with age (aged 7 months, 2 years, 3 years, and 4 years). He was also noted to have dental enamel hypoplasia and hypermobility of his small joints but no evidence of joint dislocation or herniation. He has not had any further fracture recently and currently, not on any treatment.

**Figure 2:**
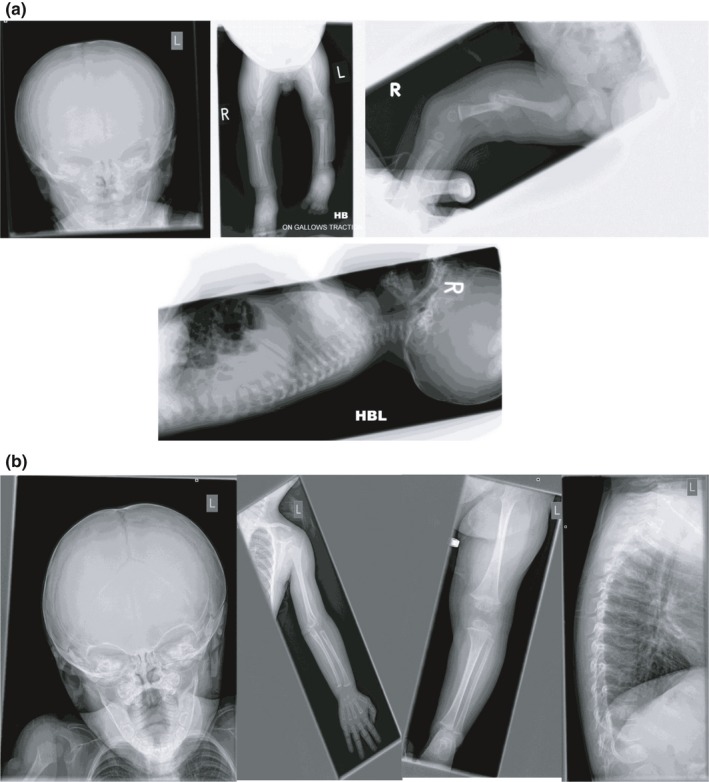
(a) Skeletal survey demonstrating osteopenia with Wormian bones, normal vertebrae in Patient 1a with evidence of fractured femur in comparison to (b) X‐rays in Patient 1b who has no evidence of OI part from osteopenia

Investigations in Patient 1a have included a skeletal survey which demonstrated osteopenia with a small number of Wormian bones in the lambdoid suture but with normal vertebrae (Figure 2). His bone biochemistry was reported normal and lumbar spine DXA showed bone mineral density (BMD) at the lower end of normal range. His bone densitometry at 7 years of age subsequently showed low forearm trabecular score with a background of normal bone profile (pQTC score −2.24 SDS with a cortical and trabecular score of −1.58SDS) and a delayed bone age by 1.5 years. His array CGH showed a 619 Kb maternally inherited Xp22.3 duplication which was predicted to be nonpathogenic. His ophthalmology review and cranial USS were normal.

**Figure 3:**
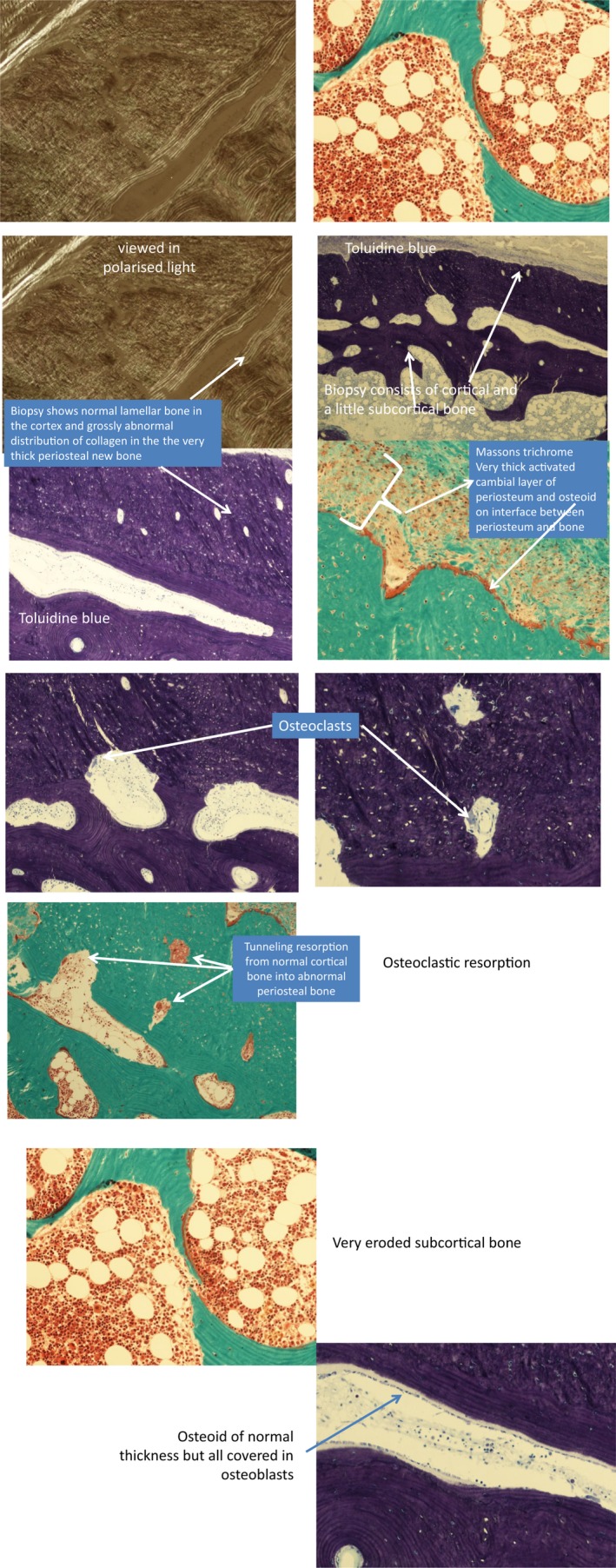
Bone biopsy undertaken at 2 years of age in Patient 1a demonstrating active new bone formation within the periosteum and increased osteoclasis within the cortex confirming advanced osteoporosis. Trabecular bone showed cortical osteopenia as a consequence of relative increase in osteoclastic over osteoblastic activity. The findings from bone biopsy were reported to be very typical of severe osteoporosis/ osteogenesis imperfecta with evidence of compensatory periosteal new bone formation to thicken the cortex but the new cortical bone deposited on the endosteal surface was also found to be significantly osteoporotic

A transiliac bone biopsy performed at 2 years of age demonstrated active new bone formation within the periosteum; bone cortices were of normal thickness but of increased porosity. The trabecular bone showed features in keeping with advanced osteoporosis. Overall, the cortical osteopenia noted was deemed to be a consequence of increased osteoclastic to osteoblastic activity within the trabecular bone presumed to be due to severe osteoporosis (Figure 3). Genetic analysis for *COL1A1/A2* identified a de novo novel in‐frame deletion in exon 2 which is described in further detail below.

**Figure 4:**
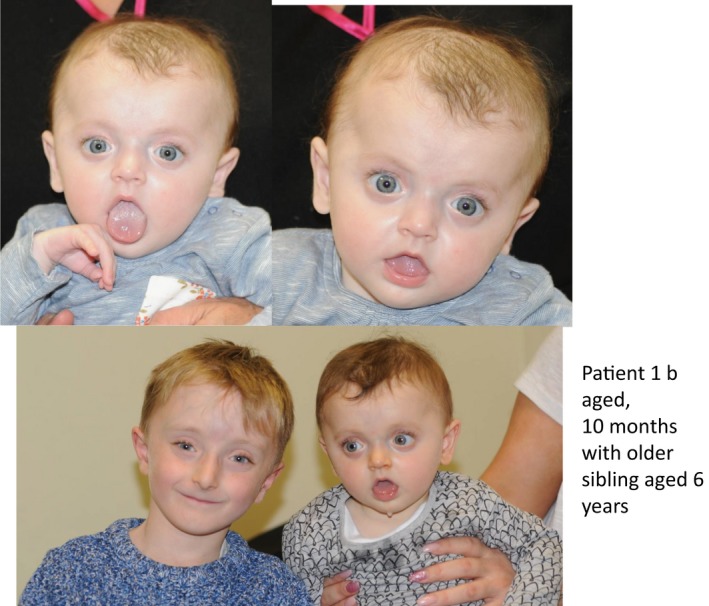
Younger sibling aged 9 months in Family 1 demonstrating strikingly similar facial dysmorphism to older brother aged 6 years

The younger sister of this patient (Patient 1b) was assessed in clinic and found to have similar facial dysmorphism as the brother and tested positive for the same familial variant indicating gonadal mosaicism in one of the parents. She was born following a normal pregnancy at 37 weeks gestation with a birth weight of 2.40 kg (0.4th centile) and was in a good condition immediately after birth. On examination at 9 months of age, she was noted to have similar facial appearance to her brother (Figure 4 showing younger sibling aged 10 months with Patient 1a aged 6 years) with blueish sclerae and premature thelarche (with normal endocrine investigations). Her growth parameters were all on the ninth centile. She has not sustained any fractures so far and her skeletal survey was reported normal. She remains under follow‐up with the Metabolic Bone team.

In view of Patient 1a clinical phenotype, fracture history, bone biopsy findings, and use of Pamidronate for treatment of his initial bone fragility, he was presumed to have a clinical diagnosis of type 1 OI. His sister was thought to have osteopenia without any fractures which may partly attributable to her younger age and closer monitoring of her bone health.

## 3 MATERIALS AND METHODS

### 3.1 Genetic analysis

Targeted gene testing for *COL1A1/A2* was performed using previously published methods in Family 1.

### 3.2 Ethical compliance

This study was performed with appropriate written consent from the family and as part of a research study approved by an ethics committee.

### 3.3 Cell culture and immunofluorescence

Primary control and patient‐derived fibroblasts (Patient 1 a) were cultured in Ham’s F10 (Life Technologies, Paisley, UK) supplemented with 12% fetal calf serum.

To investigate intracellular procollagen transport in control and patient cells, cells were grown for 48–72 hr on glass coverslips and incubated in the presence of 167 µM (50 µg/ml) ascorbic acid for 30 min prior to fixation. Cells were fixed with 4% paraformaldehyde in PBS and processed for immunofluorescence using either only anti‐collagen IαI (Novus Biologicals NB600‐408, rabbit, 1:2,000) for analysis of the extracellular collagen, or anti‐collagen IαI in combination with the COPII marker anti‐Sec31A (BD Biosciences 612,350, mouse, 1:1,000) or the *cis*‐Golgi marker anti‐GM130 (BD Biosciences 610,823, mouse, 1:1,000) and imaged by confocal microscopy as described previously (McCaughey et al., 2016) using an Alexa‐Fluor‐anti‐rabbit‐568‐conjugated (Thermo Fisher Scientific) and/or Alexa‐Fluor‐anti‐mouse‐488‐conjugated secondary antibody, Prolong Diamond with DAPI for mounting and a Leica SP5 for analysis and image acquisition.

### 3.4 Collagen secretion assay by Western Blot

For semiquantitative analysis of COL1a1 levels in control and patient cells, cells were seeded confluent into a 6 cm dish and grown for 24 hr, following incubation in serum‐free media either without or supplemented with 167 µM (50 µg/ml) ascorbate for 24 hr at 37°C and 5% CO_2_. The medium was collected and the cells incubated in lysis buffer containing 50 mM Tris‐HCl, 150 mM NaCl, 1% (v/v) triton‐X‐100, 1% (v/v) protease inhibitor cocktail (Calbiochem) at pH 7.4 for 15 min on ice. Protein fractions of medium and lysate were centrifuged at 13,500 rpm at 4°C for 10 min. The cell pellet was discarded. The supernatant was denatured and run under reducing conditions on a 3%–8% Tris‐Acetate precast gels (NuPAGE ®) for 75 min at 150 V in Tris‐Acetate running buffer supplemented with antioxidant. Transfer of protein bands onto a nitrocellulose membrane was performed at 300 mA for 106 min. The membrane was blocked using 5% (w/v) milk powder in TBST overnight at 4°C and incubated with antibodies against collagen IαI (Novus Biologicals NB600‐408; rabbit, 1:1,000) and DIC74.1 (Merck MAB1618, mouse, 1:1,000) as loading control for 1.5 hr at RT. After repeated rinsing with TBST, the membrane was incubated for 1.5 hr at RT with HRP‐conjugated antibodies diluted in the blocking solution (1:5,000) against mouse and rabbit, respectively. The wash step was repeated, and detection was performed using Promega WB‐ECL reaction reagents and autoradiography films with overnight exposure and subsequent development.

### 3.5 Cell extraction and harvest of cell derived matrix

Cells were rinsed twice with PBS prior to incubation with 20 mM ammonium hydroxide in PBS for 5 min at room temperature and vigorous shaking on a rocker. Detached cells were rinsed via three washes with ddH_2_O. Plates containing the cell‐derived matrix were incubated with DNAseI at 10 µg ∙ ml^‐1^ for 30 min at 37°C followed by three washes with ddH_2_O. Matrix proteins were covered with 5% acetic acid and incubated at 4°C overnight. Matrix proteins were extracted with a cell scraper from the dish after addition of matrix buffer containing 125 mM Tris‐HCl, pH 6.8, 0.1% SDS, 10% glycerol, 1% DDT and a protease inhibitor cocktail (Calbiochem). Protein extracts and acetic acid fractions were combined into a polypropylene ultracentrifugation tube and five volumes of prechilled acetone (−20°C) were added to the samples and proteins were precipitated overnight at −20°C. Proteins were pelleted via a Sorvall Evolution centrifuge at 15,000 *× g* for 10 min at 4°C. The supernatant was decanted and the protein pellet air‐dried at room temperature. Samples were resuspended in matrix buffer and the protein concentration was determined using NanoDrop at 280 nm. Samples for subsequent proteomics analysis contained 100 µg of protein extract at 1–2 µg/µl.

### 3.6 Proteomic analysis

For TMT Labeling and high pH reversed‐phase chromatography, the samples were labeled with tandem mass tag (TMT) multiplex reagents according to the manufacturer’s protocol (Thermo Fisher Scientific, Loughborough, UK) and the labeled samples were pooled. The pooled sample was then desalted using a SepPak cartridge according to the manufacturer’s instructions (Waters, Milford, Massachusetts, USA). Eluate from the SepPak cartridge was evaporated to dryness and resuspended in buffer A (20 mM ammonium hydroxide, pH 10) prior to fractionation by high pH reversed‐phase chromatography using an Ultimate 3,000 liquid chromatography system (Thermo Fisher Scientific). In brief, the sample was loaded onto an XBridge BEH C18 Column (130 Å, 3.5 µm, 2.1 mm × 150 mm, Waters, UK) in buffer A and peptides eluted with an increasing gradient of buffer B (20 mM ammonium hydroxide in acetonitrile, pH 10) from 0% to 95% over 60 min. The resulting fractions were evaporated to dryness and resuspended in 1% formic acid prior to analysis by nano‐LC MSMS using an Orbitrap Fusion Tribrid mass spectrometer (Thermo Fisher Scientific).

### 3.7 Semiquantification of extracellular collagen levels

In order to estimate the relative amount of secreted collagen type I, the corrected total cell fluorescence (CTCF) per field of view of four to five randomly picked areas per sample (control and patient cells) was calculated according to (McCloy et al., 2014) where CTCF = [integrated density − (area of selected cell × mean background fluorescence)]. Subsequently, the ratio of the patient sample CTCFs and the mean of the control sample CTCFs were calculated. The resulting ratios originate from three independent experiments.

### 3.8 Nano‐LC mass spectrometry

High pH RP fractions were further fractionated using an Ultimate 3,000 nano‐LC system in line with an Orbitrap Fusion Tribrid mass spectrometer (Thermo Fisher Scientific). In brief, peptides in 1% (vol/vol) formic acid were injected onto an Acclaim PepMap C18 nano‐trap column (Thermo Fisher Scientific). After washing with 0.5% (vol/vol) acetonitrile 0.1% (vol/vol) formic acid, peptides were resolved on a 250 mm × 75 μm Acclaim PepMap C18 reverse phase analytical column (Thermo Fisher Scientific) over a 150 min organic gradient, using seven gradient segments (1%–6% solvent B over 1 min, 6%–15% B over 58 min, 15%–32% B over 58 min, 32%–40% B over 5 min, 40%–90% B over 1 min, held at 90% B for 6 min and then reduced to 1% B over 1 min) with a flow rate of 300 nl/min. Solvent A was 0.1% formic acid and Solvent B was aqueous 80% acetonitrile in 0.1% formic acid. Peptides were ionized by nano‐electrospray ionization at 2.0 kV using a stainless steel emitter with an internal diameter of 30 μm (Thermo Fisher Scientific) and a capillary temperature of 275°C.

All spectra were acquired using an Orbitrap Fusion Tribrid mass spectrometer controlled by Xcalibur 2.0 software (Thermo Fisher Scientific) and operated in data‐dependent acquisition mode using an SPS‐MS3 workflow. FTMS1 spectra were collected at a resolution of 120,000, with an automatic gain control (AGC) target of 400,000 and a max injection time of 100 ms. Precursors were filtered with an intensity range from 5,000 to 1E20, according to charge state (to include charge states 2–6) and with monoisotopic precursor selection. Previously interrogated precursors were excluded using a dynamic window (60 s ± 10 ppm). The MS2 precursors were isolated with a quadrupole mass filter set to a width of 1.2 m/z. ITMS2 spectra were collected with an AGC target of 10,000, max injection time of 70 ms and CID collision energy of 35%.

For FTMS3 analysis, the Orbitrap was operated at 30,000 resolution with an AGC target of 50,000 and a max injection time of 105 ms. Precursors were fragmented by high energy collision dissociation (HCD) at a normalized collision energy of 55% to ensure maximal TMT reporter ion yield. Synchronous Precursor Selection (SPS) was enabled to include up to 5 MS2 fragment ions in the FTMS3 scan.

### 3.9 Data analysis

The raw data files were processed and quantified using Proteome Discoverer software v2.1 (Thermo Fisher Scientific) and searched against the UniProt Human database (140,000 entries) using the SEQUEST algorithm. Peptide precursor mass tolerance was set at 10 ppm, and MS/MS tolerance was set at 0.6 Da. Search criteria included oxidation of methionine and proline (+15.9949) as a variable modification and carbamidomethylation of cysteine (+57.0214) and the addition of the TMT mass tag (+229.163) to peptide N‐termini and lysine as fixed modifications. Searches were performed with full tryptic digestion and a maximum of three missed cleavage sites was allowed. The reverse database search option was enabled and the data were filtered to satisfy false discovery rate (FDR) of 5%.

## 4 RESULTS

### 4.1 Family 1

#### 4.1.1 Genetic analysis

Targeted testing for *COL1A1/A2* identified a de novo* COL1A1* c.206_208delTGT, p.Leu69del variant which results in deletion of one amino acid residue on the N‐propeptide of pro*α*1(1) chain of type 1 collagen. This variant has not been reported in the OI variant database. Triple helix in‐frame deletions of *COL1A1* have been reported previously but are not common, and typically result in a more severe phenotype. This variant remained a VUS until further functional studies were undertaken to prove pathogenicity.

#### 4.1.2 Collagen processing and proteomics

**Figure 5:**
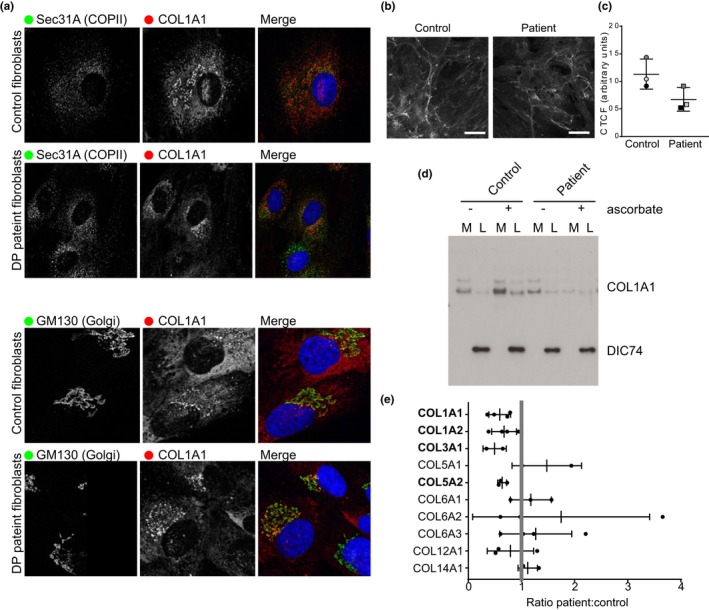
Collagen secretion defects in fibroblasts from patient 1a. (a) Control or patient fibroblasts incubated for 30 min to promote procollagen secretion were immunolabeled to detect COL1A1 (red) and in green, either Sec31A (to mark COPII‐coated ER exit sites) or GM130 (to mark the Golgi apparatus). (b) Extracellular collagen matrix was labeled to detect COL1A1. (c) Quantification of the mean Corrected Total Cell Fluorescence (CTCF) for COL1A1. Points show mean intensity from three independent experiments. Bars show standard deviation. (d) Immunoblot for COL1A1 in media (M) or lysates (L) of control or patient fibroblasts incubated in the presence of absence of ascorbate. (e) Unbiased proteomics was used to measure extracellular matrix collagens secreted from control or patient fibroblasts. Results from three independent experiments are shown. A ratio <1 indicates reduced collagen secretion from patient cells

Figure 5 demonstrates experimental result from fibroblasts in Patient 1a. Results from three different experiments demonstrate the *COL1A1* c.206_208delTGT, p.Leu69del variant causing a substantial defect to collagen extracellular matrix assembly.

Figure 5a shows that localization of intracellular *COL1A1* in patient cells was normal compared to control samples. In the presence of ascorbate *COL1A1* localizes to the endoplasmic reticulum and in higher concentration to areas of the Golgi apparatus. Similarly, the morphology of *COL1A1* in the extracellular matrix of patient cells is similar compared to control cells, aside from patches of high abundance in patient samples. The overall abundance of *COL1A1* appears to be lower in the patient sample by immunofluorescence (Figure 5B). This result is further supported by the protein levels detected by immunoblotting (Figure 5C), showing lower COL1A1 levels compared to control samples. Furthermore, lysates of patient‐derived cells and medium from these cells do not contain increased *COL1A1* following addition of ascorbate, as seen for the control sample. Proteomics data revealed a consistent decrease in *COL1A1*, *COL1A2*, *COL3A1,* and *COL5A2* in the cell‐derived extracellular matrix of patient cells compared to the controls. Collagens 3 and 5 are both interactors with collagen type I in the extracellular matrix. The interdependence of collagen isotypes has been described previously. It is known that dermal collagen fibrils contain both type I and type III collagens (Fleischmajer, MacDonald, Perlish, Burgeson, & Fisher, 1990) and that type I and type V collagens can also be found in the same fibrils (Birk, Fitch, Babiarz, & Linsenmayer, 1988). Expression of procollagens I and III can also be coregulated, for example, by glucocorticoid signaling (Walsh, LeLeiko, & Sterling, 1987). This is consistent with our findings in which the secretion and assembly of multiple collagen isotypes is reduced.

## 5 DISCUSSION

In Clinical Genetics practice, we currently undertake diagnostic genetic testing for a whole host of genetic conditions to confirm diagnosis, clarify prognosis, inform recurrence risks, and provide options for prenatal genetic testing. However, with increased access to genetic testing, we are identifying variants of unclassified significance, also referred to as VUS where the pathogenicity of the variant is not certain. This is becoming more of an issue with increased access to next‐generation sequencing technologies and testing of more patients with a given genetic condition. Currently, when we identify an unclassified variant from genetic testing, apart from segregation studies to identify if the variant tracks with the phenotype in the family and awaiting further knowledge, there is nothing further we can offer in the routine diagnostic setting. This affects decisions regarding treatment, unable to clarify diagnosis or provide informed recurrence risks; hence, there is a clear unmet need.

In our center, we see a large cohort of patients with OI and undertake targeted genetic analyses in this group. Based on further research into collagen processing and extracellular matrix assembly of collagen, we plan to develop a diagnostic pathway to better identify and clarify the pathogenicity of these variants in order to provide patients with a more definitive genetic diagnosis, accurate recurrence risks, and options of prenatal testing.

Data presented here show that with a VUS in *COL1A1*, we have been able to clarify pathogenicity by undertaking detailed phenotyping of extended family; segregation studies in family members (affected vs. unaffected) and functional studies on cultured fibroblasts. With projects underway nationally including the 100,000 Genomes Project (https://www.genomicsengland.co.uk/); VUS will become more of an issue with increased access to genetic testing.

Using one such family with *COL1A1* VUS shown here, we are able to confirm that this variant is pathogenic. The reduction in secretion and assembly of type 1 collagen is consistent with a mild OI presentation in Patient 1a. We have used light microscopy to visualize the structure of the extracellular matrix and intracellular collagen. We used TMT to label individual samples with isobaric tags and quantify abundance of key proteins by LC‐MS/MS. Patient 1a has a more severe phenotype compared to his sibling which may simply reflect the fact that she is younger and been more closely monitored clinically although, it is also possible that there are genetic modifiers which is altering the clinical severity in these siblings. Phenotypic variability even within the same family is a well‐recognized feature in OI and studies are ongoing to evaluate why this may be the case in large multi‐generational families with the same genotype but variable severity.

Cells from Patient 1a deposit fewer collagen fibrils that often form more densely packed areas. These data show that pathogenic variant in procollagen lead to defects in the assembly and deposition, not only of collagen but of many other ECM proteins. Data presented here shows that detailed phenotyping in addition to segregation studies and cell studies focussing on light microscopy, quantitative proteomics, and analysis of procollagen trafficking and secretory pathway function in the workflow can help clarify the pathogenicity of such variants.

Our analysis of fibroblasts from Patient 1a with a VUS in *COL1A1* also provides mechanistic insight into key questions of fundamental cell biology. Our recent work has led us to hypothesize that the composition of the ECM dictates the organization and function of the secretory pathway by feedback control. Our hypothesis is that these are linked directly such that not only does secretion directly impact the composition of the ECM but equally the composition of the ECM dictates the composition of the Golgi via a feedback mechanism.

For OI families, this opens the option of testing of unaffected family members to clarify risk, prenatal genetic testing, follow‐up arrangements for the unaffected relatives, thus having a positive impact for the families as demonstrated clearly in the family reported here.

## 6 CONCLUSIONS

With advances in genetic testing and access to whole exome/ genome sequencing technologies, VUS are an increasing finding in Clinical Genetics practice. This is particularly relevant for OI where there are treatment options available and management decisions are made on the basis of genetic testing results in conjunction with clinical and radiological evidence. This is also true in nonaccidental injury scenario where access to genetic testing is increasing in order to ascertain/ refute diagnosis of OI. Having a clinical pathway for clarifying diagnosis of such VUS identified in type 1 Collagen genes in OI which goes beyond the realms of segregation studies and detailed phenotyping is extremely useful in this patient group. Studies such as this will aid in understanding more about underlying disease mechanism and provide insights into collagen secretion advancing us further in translating these discoveries into patient benefit.

## CONFLICT OF INTEREST

None of the authors have any conflict of interest to declare.

## AUTHORS’ CONTRIBUTION

MB planned the study, recruited all the patients and undertook further studies, and wrote the manuscript. EH and MS were involved in patient care and phenotyping. JM and DJS undertook fibroblast analyses and proteomics study. All authors reviewed and contributed to the manuscript.
